# Supplementary material for: Integrated analysis of ischemic stroke datasets revealed sex and age difference in anti-stroke targets
Source: PeerJ. 2016 Sep 15;4:e2470. doi: 10.7717/peerj.2470 (PMC5028792; doi:10.7717/peerj.2470)
Supplement: Supplemental Information 2 — Supplementary Tables and Figures. [file peerj-04-2470-s002.docx]

**Supplementary Table 1.** Details of the ischemic stroke datasets.

|  | **GSE16561** | **GSE22255** | **GSE37587*** |
| --- | --- | --- | --- |
| Contributor(s) | Barr TL et al., 2009 | Krug T et al., 2010 | Barr TL et al, 2012 |
| Samples |  |  |  |
| Patients | 39 | 20 | 34 |
| Controls | 24 | 20 | 0 |
| Cell Type | Peripheral Blood Mononuclear Cells | Peripheral Blood Mononuclear Cells | Peripheral Blood Mononuclear Cells |
| Platform | GPL6883: Illumina HumanRef-8 v3.0 Expression BeadChip | GPL570: Affymetrix Human Genome U133 Plus 2.0 Array | GPL6883: Illumina HumanRef-8 v3.0 Expression BeadChip |
| Links | http://www.ncbi.nlm.nih.gov/geo/query/acc.cgi?acc=GSE16561 | http://www.ncbi.nlm.nih.gov/geo/query/acc.cgi?acc=GSE22255 | http://www.ncbi.nlm.nih.gov/geo/query/acc.cgi?acc=GSE37587 |

* This dataset was used to verify the results.

**Supplementary Table 2.** Age distribution of ischemic stroke patients and controls.

| **Group** | **Number (%)** | **Age (mean ± SD)** | **P^1^** |
| --- | --- | --- | --- |
| Total |  |  |  |
| Patients | 59 (57.3) | 68.7 ± 14.2 | < 0.001 |
| Controls | 44 (42.7) | 59.4 ± 10.2 |  |
| Male |  |  |  |
| Patients | 27 (57.4) | 68.4 ± 13.5 | 0.033 |
| Controls | 20 (42.6) | 60.9 ± 9.9 |  |
| Female |  |  |  |
| Patients | 32 (57.1) | 69.0 ± 15.0 | 0.002 |
| Controls | 24 (42.9) | 58.1 ± 10.5 |  |
| Old |  |  |  |
| Patients | 41 (65.1) | 76.6 ± 8.7 | < 0.001 |
| Controls | 22 (34.9) | 68.1 ± 5.5 |  |
| Young |  |  |  |
| Patients | 18 (45.0) | 50.7 ± 4.5 | 0.959 |
| Controls | 22 (55.0) | 50.6 ± 4.7 |  |

^1^ Use Student's t-test to compare the age between patients and controls.


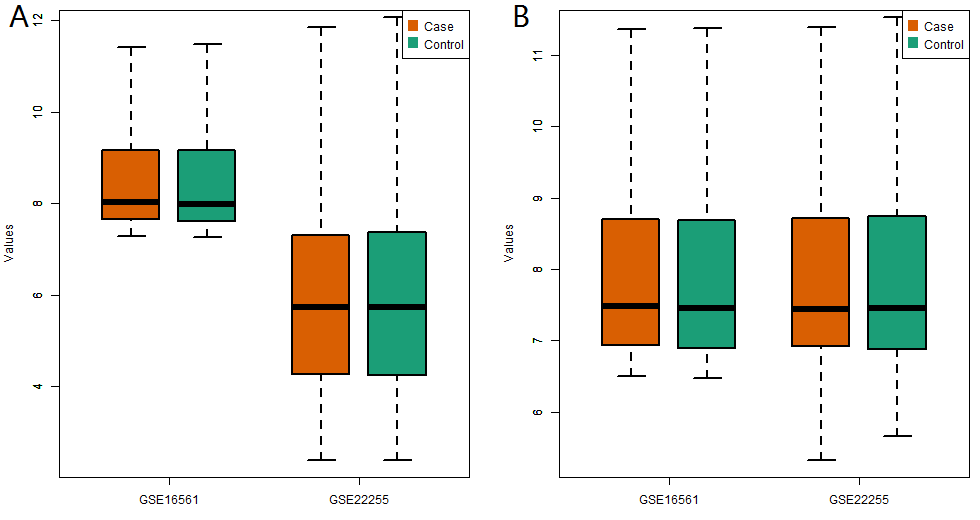


**Supplementary Figure 1.** Value distribution of GSE16561 and GSE22255. Panel **A** showed the distribution of RMA processed gene expression values of ischemic stroke datasets. There was a large deviation in the distribution of gene expression values in the two datasets. Panel **B** showed the distribution of global renormalized (after RMA processed) gene expression values of ischemic stroke datasets. The distribution of gene expression values in the two datasets had a relatively consistent range.


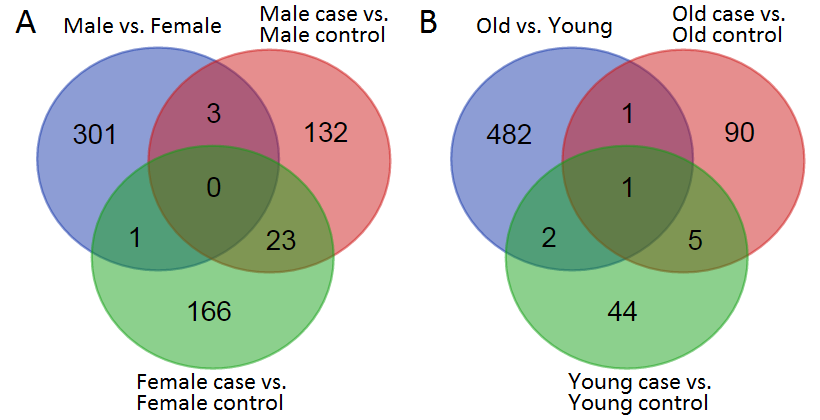


**Supplementary Figure 2.** Differentially expressed genes validation in male, female, old and young group. Panel **A** was the Venn diagram of differentially expressed genes in male vs. female, male case vs. male control, and female case vs. female control. There were 13 overlapped genes in male vs. female and other groups (PPP1R15B, ZFP36L1, SAMSN1, ACSL1, IL8, DPYSL5, G0S2, BCL2A1, TLR1, BNIP3L, APOBEC3A, S100A12 and HMGB2). Panel **B** was the Venn diagram of differentially expressed genes in old vs. young, old case vs. old control, and young case vs. young control. There were 19 overlapped genes in old vs. young and other groups (ANXA3, OLFM4, CEACAM8, IQGAP1, HSPE1, HSPA1A, S100A12, ARG1, MMP9, KRT1, ABCA1, ACSL1, CXCL1, NFIL3, IL8, PTGS2, APOBEC3A, G0S2 and BCL2A1).


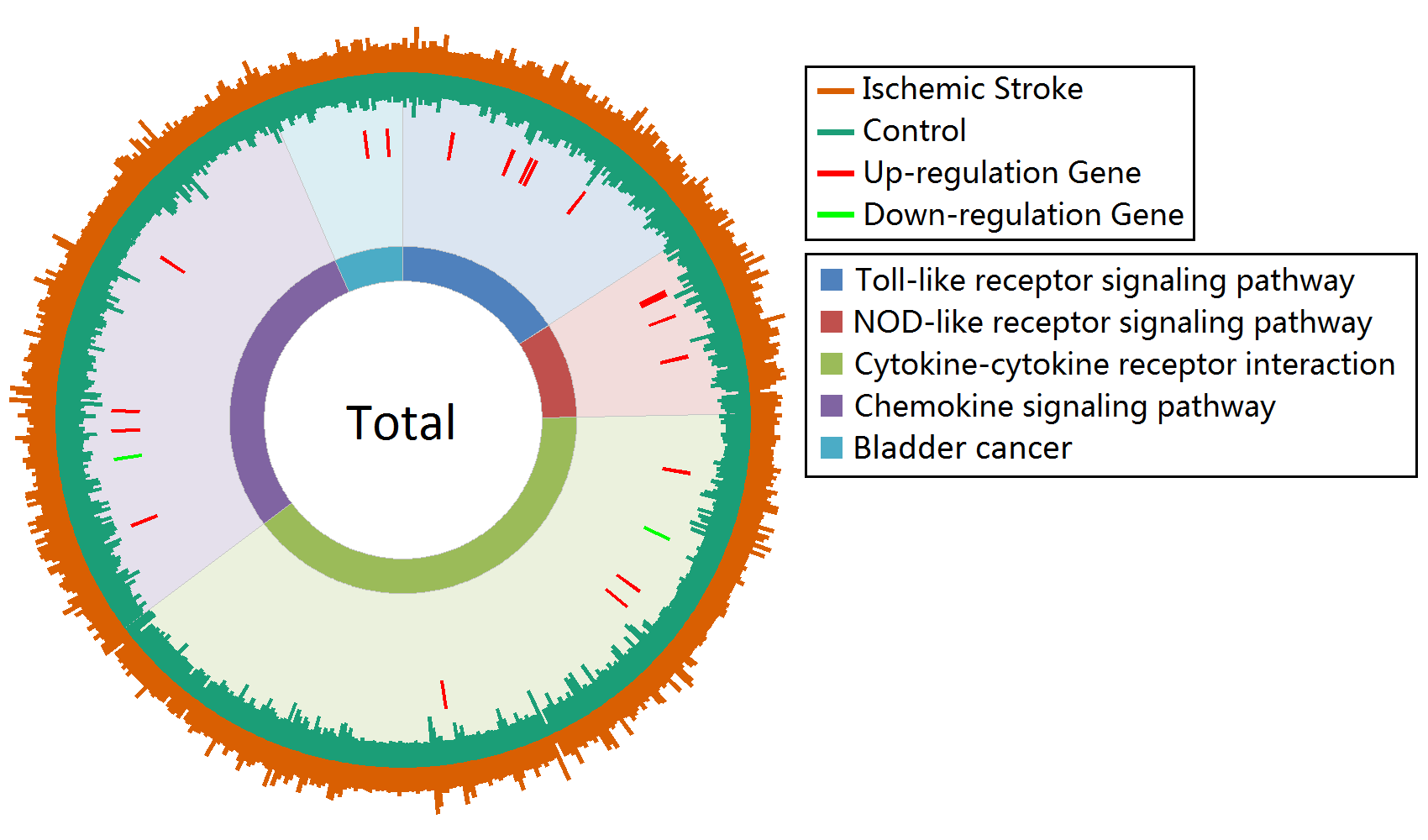


**Supplementary Figure 3.** Gene expression profiles of enrichment pathways in total gruop. Pathways are represented by different colors.


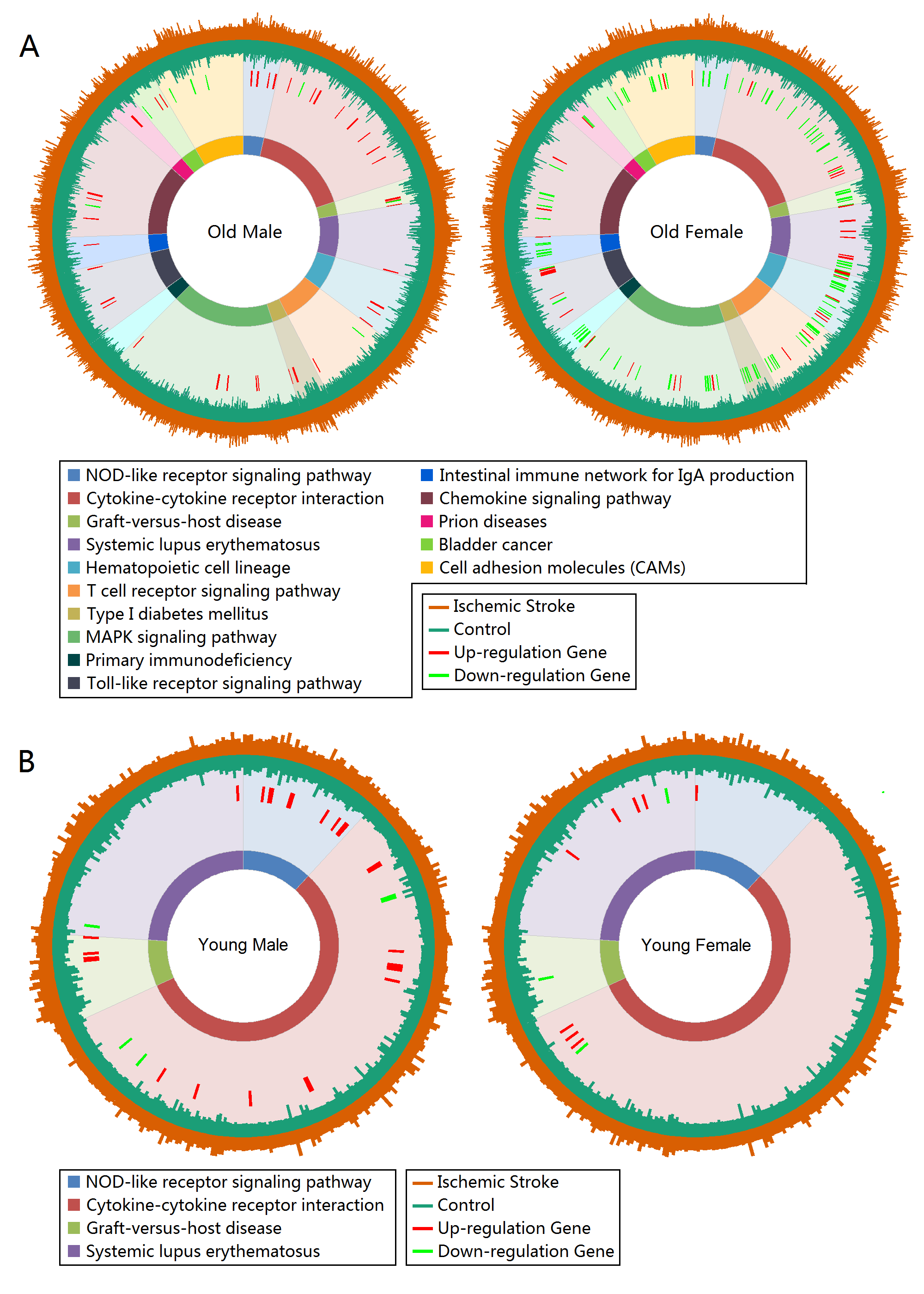


**Supplementary Figure 4.** The role of age in sex dimorphism of ischemic stroke. Panel **A** showed the pathways and expression profiles in old male and old female group. Panel **B** showed the pathways and expression profiles in young male and young female group. Pathways are represented by different colors.


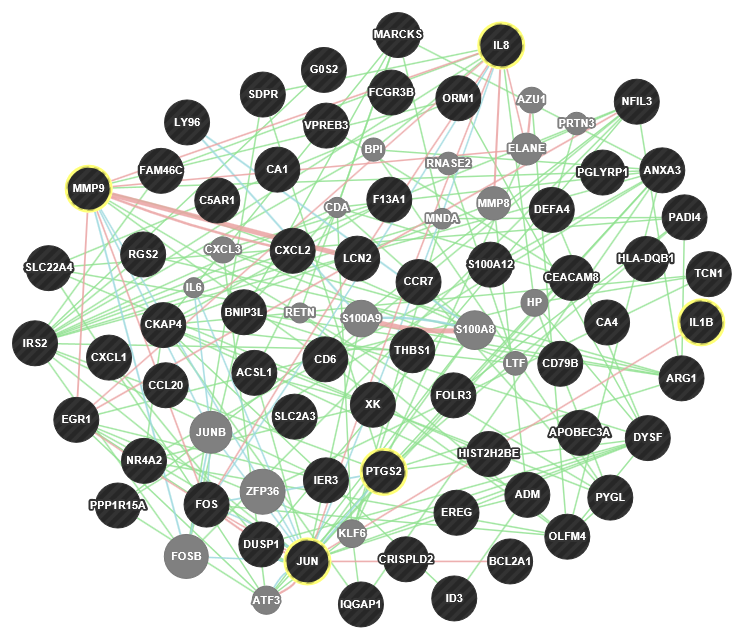


**Supplementary Figure 5.** Functional interaction network in total group. Physical interactions, genetics interactions and pathways between two proteins represent by red, green and blue color, respectively. The stroke targets showed in yellow cycle.


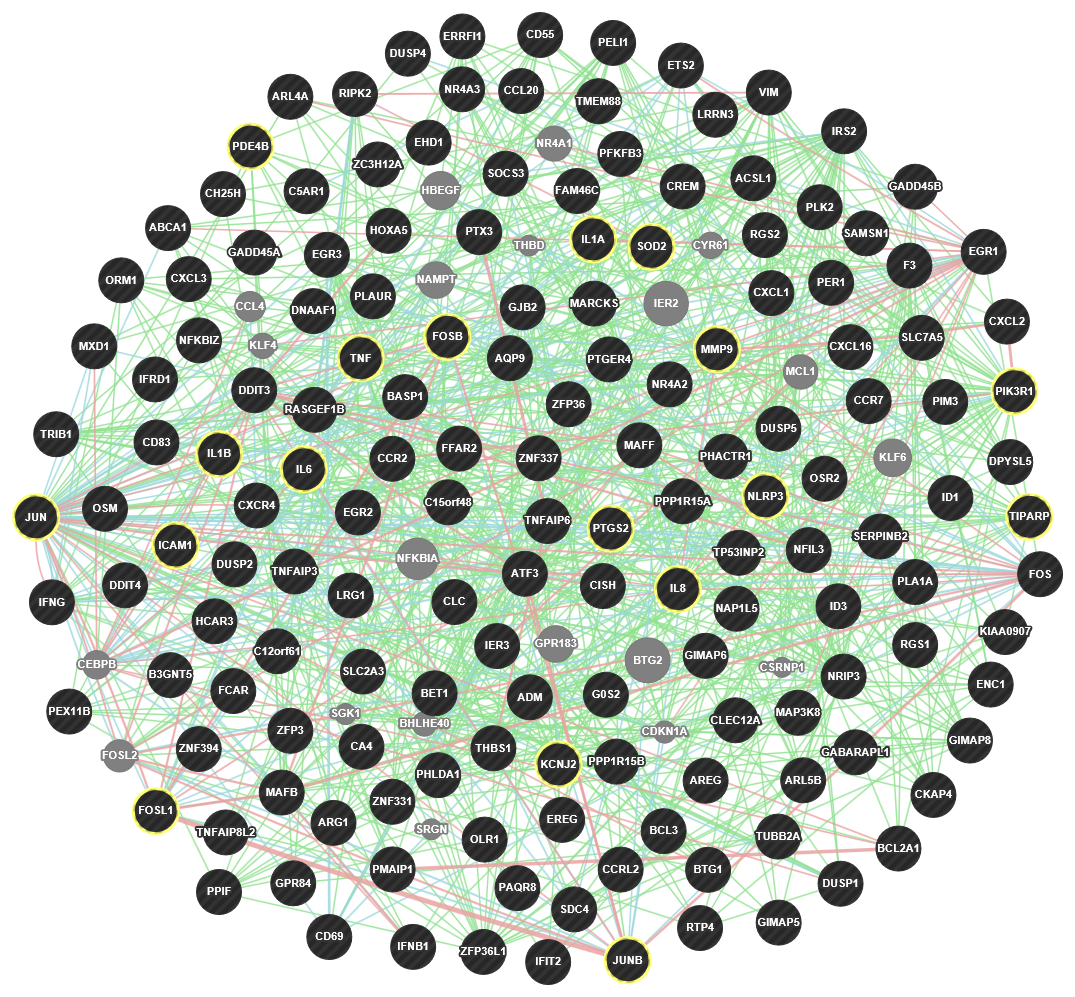


**Supplementary Figure 6.** Functional interaction network in male group. Physical interactions, genetics interactions and pathways between two proteins represent by red, green and blue color, respectively. The stroke targets showed in yellow cycle.


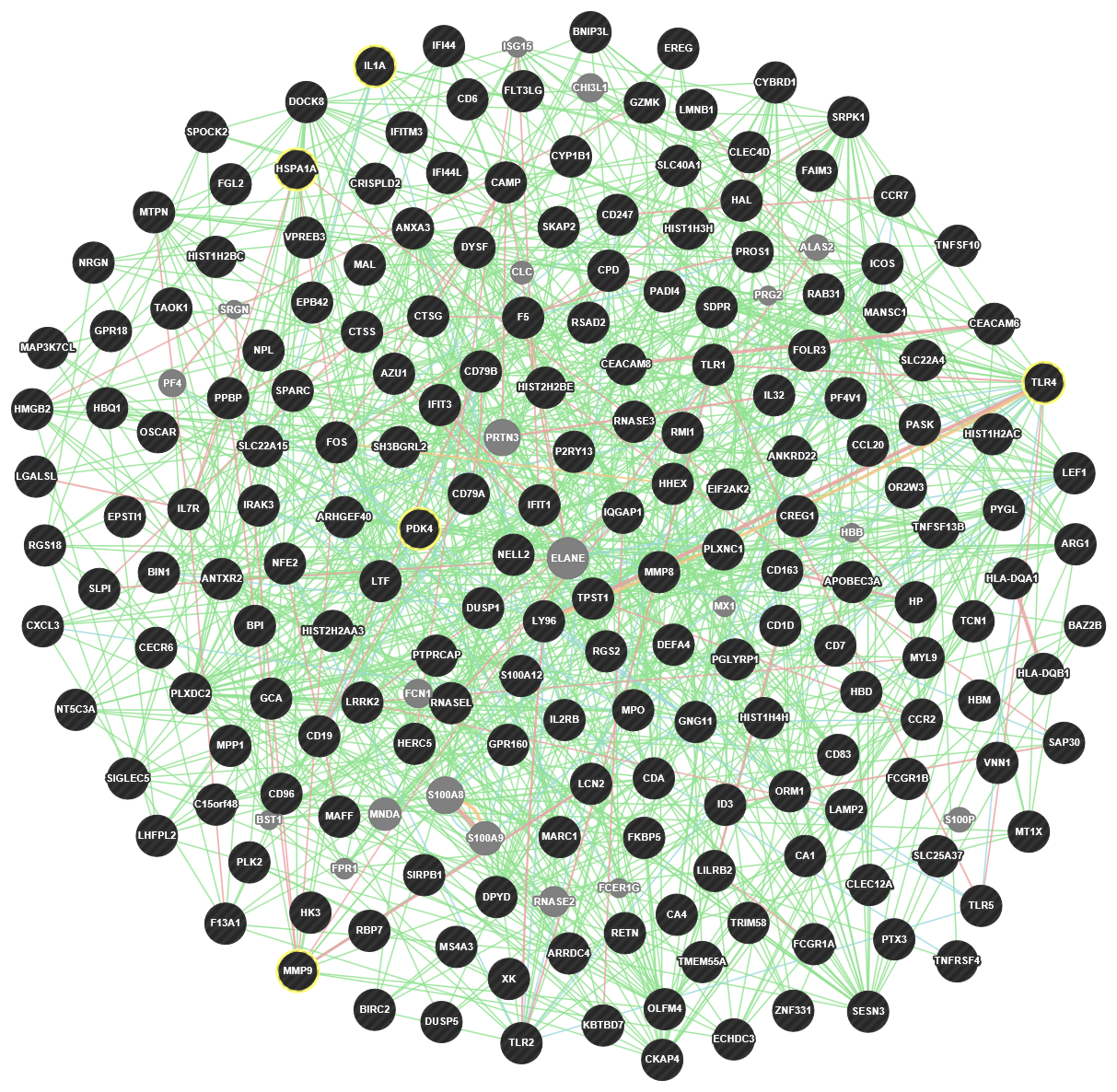


**Supplementary Figure 7.** Functional interaction network in female group. Physical interactions, genetics interactions and pathways between two proteins represent by red, green and blue color, respectively. The stroke targets showed in yellow cycle.


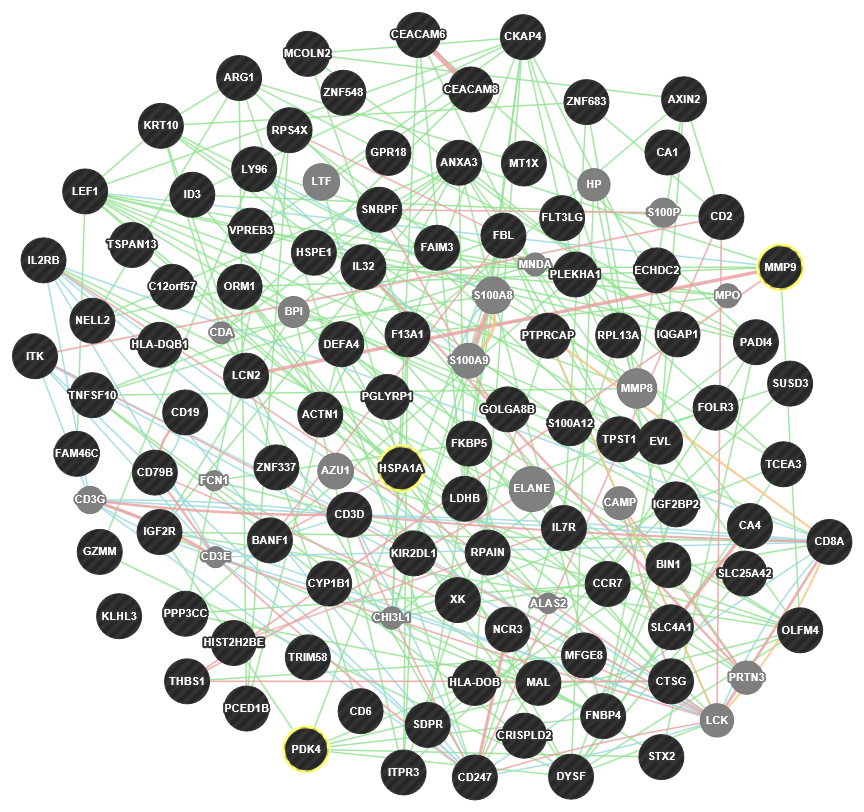


**Supplementary Figure 8.** Functional interaction network in old group. Physical interactions, genetics interactions and pathways between two proteins represent by red, green and blue color, respectively. The stroke targets showed in yellow cycle.


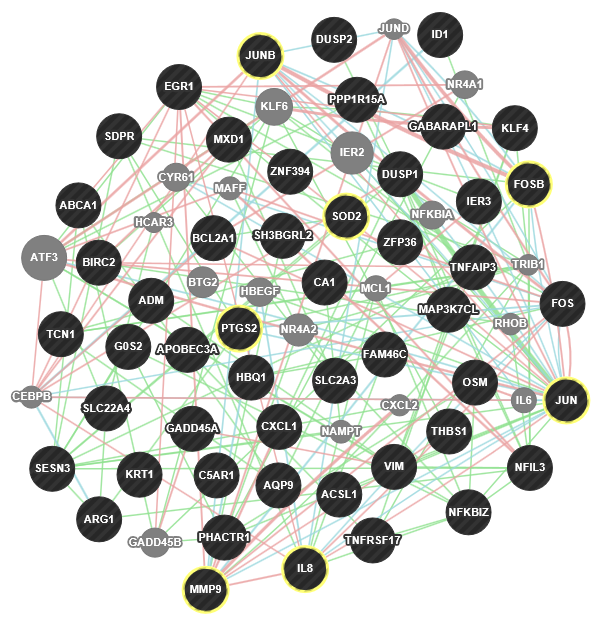


**Supplementary Figure 9.** Functional interaction network in young group. Physical interactions, genetics interactions and pathways between two proteins represent by red, green and blue color, respectively. The stroke targets showed in yellow cycle.


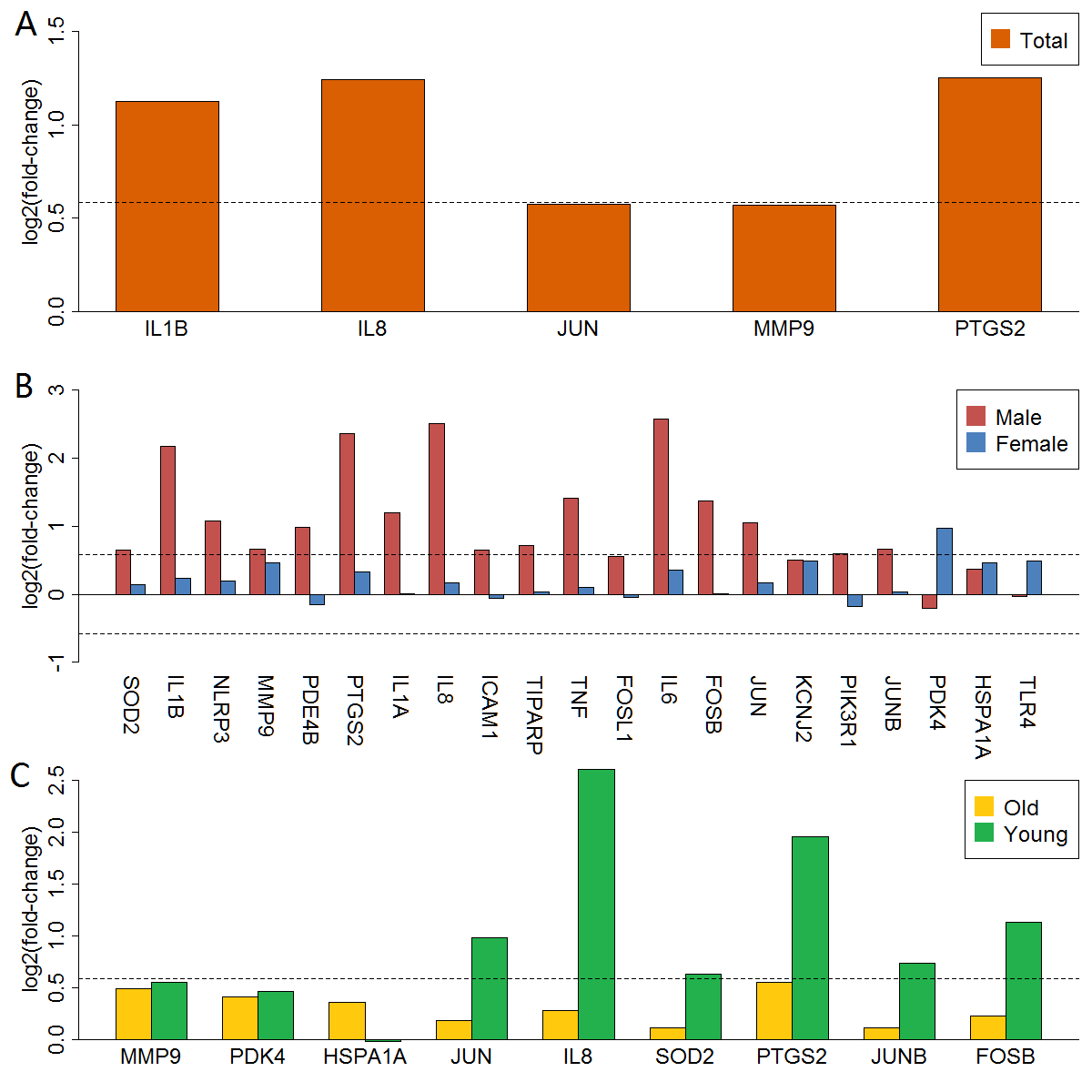


**Supplementary Figure 10.** Validation of mapped anti-stroke target genes. Panel **A** showed the logFC in total group. Panel **B** showed the logFC in male and female group. Panel **C** showed the logFC in old and young group. The horizontal dashed lines represent the logFC cutoff of the up- and down-regulated genes.


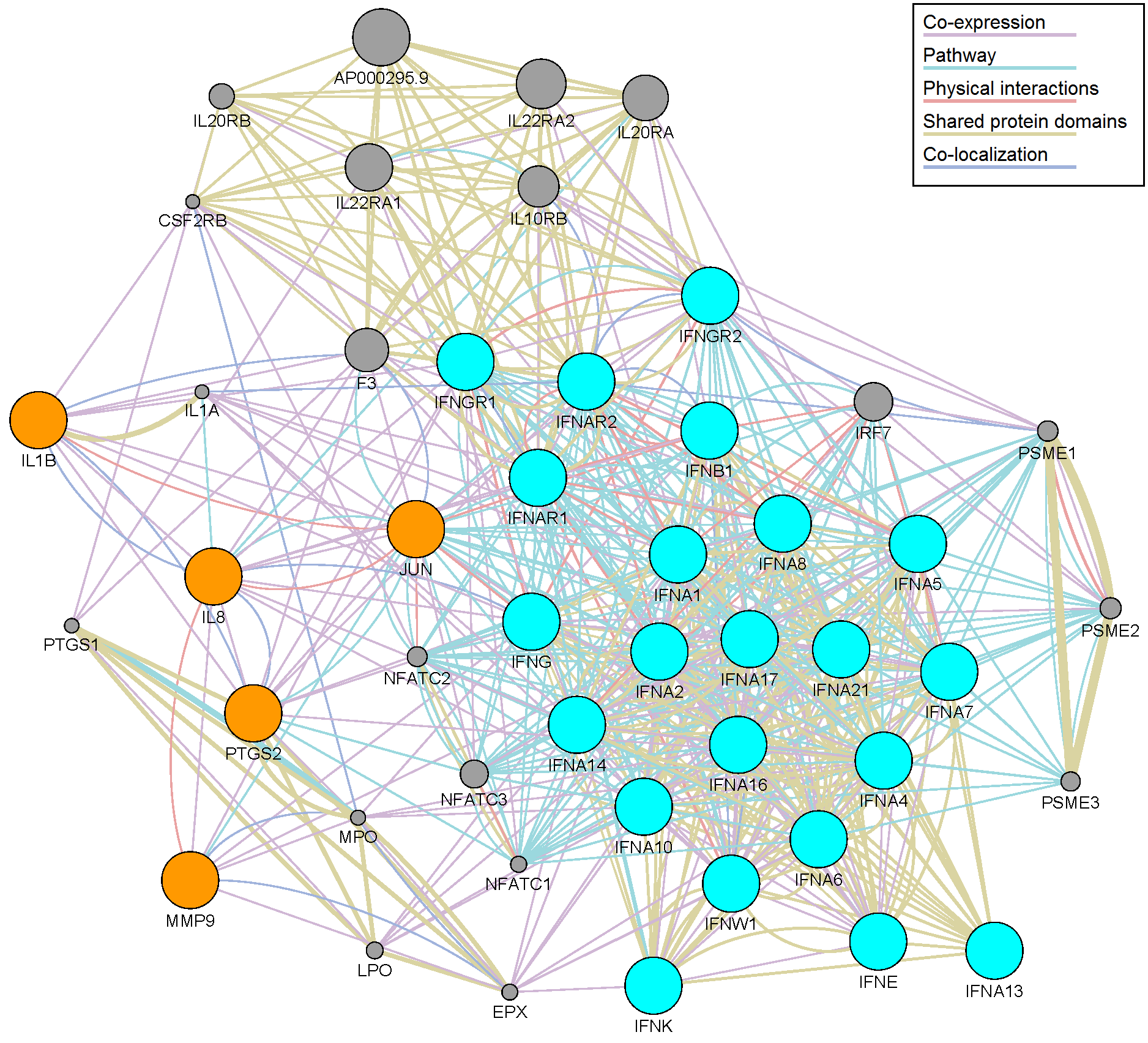


**Supplementary Figure 11.** Connection of interferon signaling genes and anti-stroke target genes in the total group. The cyan cycle represented the interferon signaling genes, and the orange cycle represented the anti-stroke target genes.


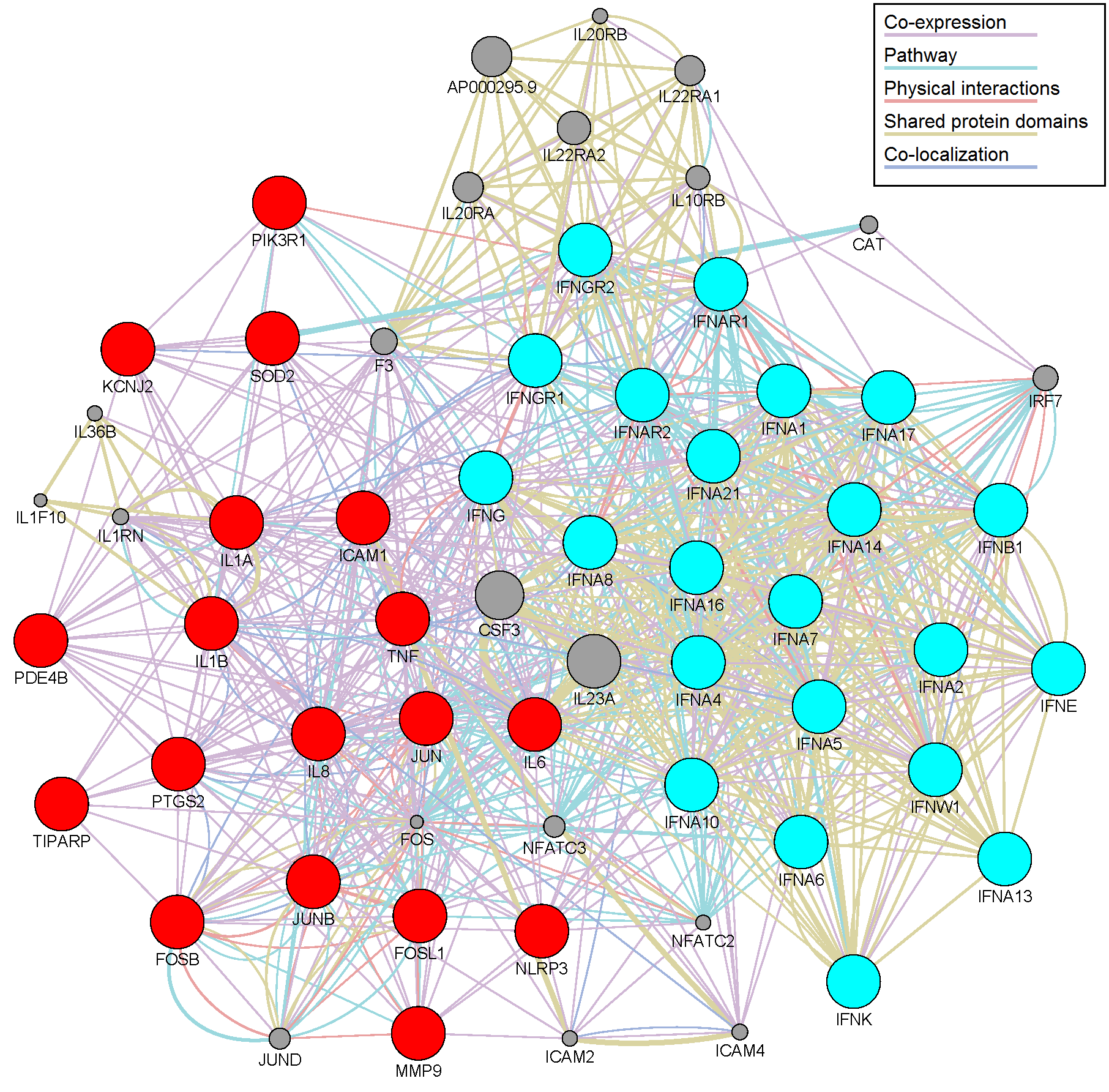


**Supplementary Figure 12.** Connection of interferon signaling genes and anti-stroke target genes in the male group. The cyan cycle represented the interferon signaling genes, and the red cycle represented the anti-stroke target genes.


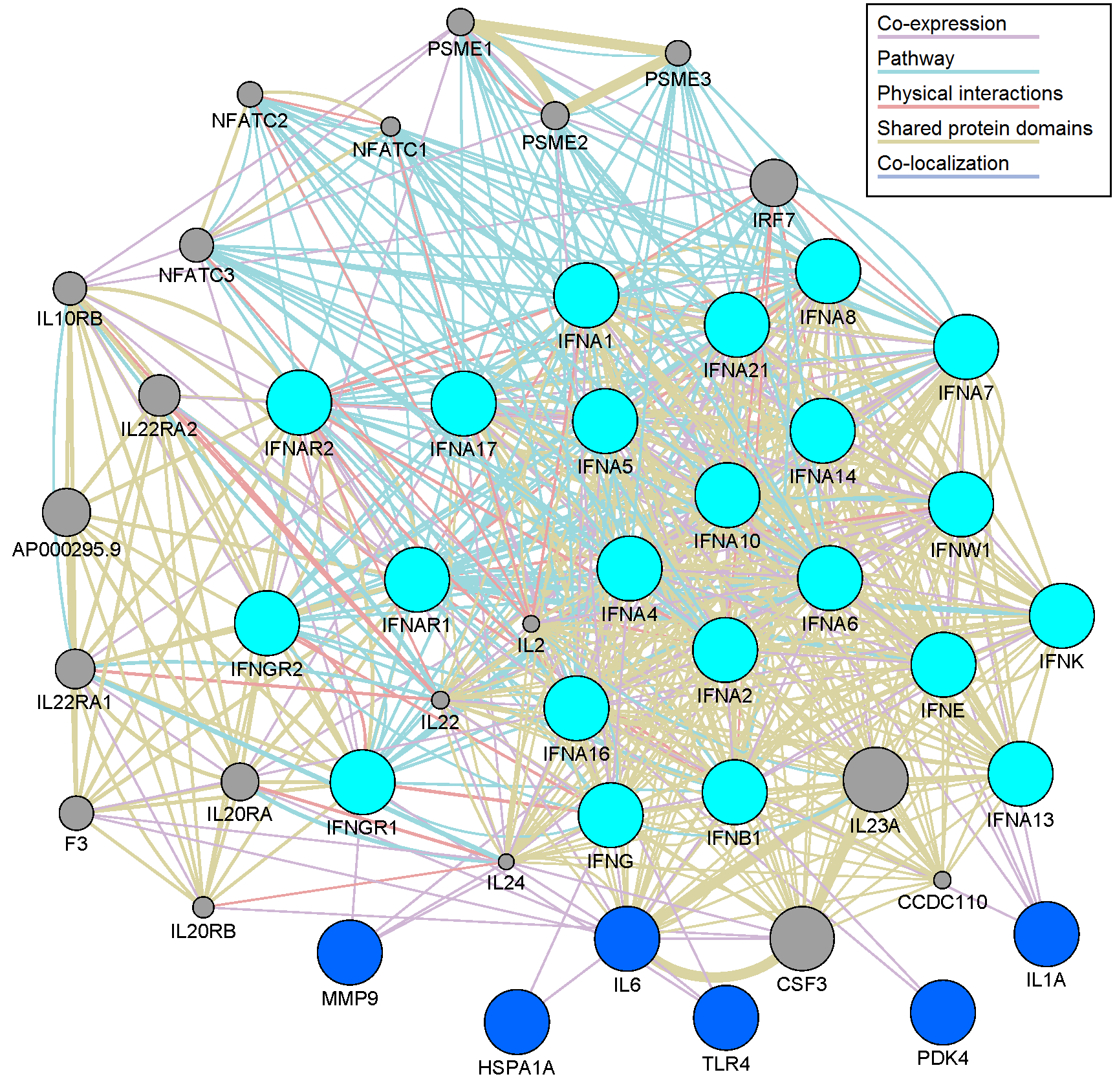


**Supplementary Figure 13.** Connection of interferon signaling genes and anti-stroke target genes in the female group. The cyan cycle represented the interferon signaling genes, and the blue cycle represented the anti-stroke target genes.


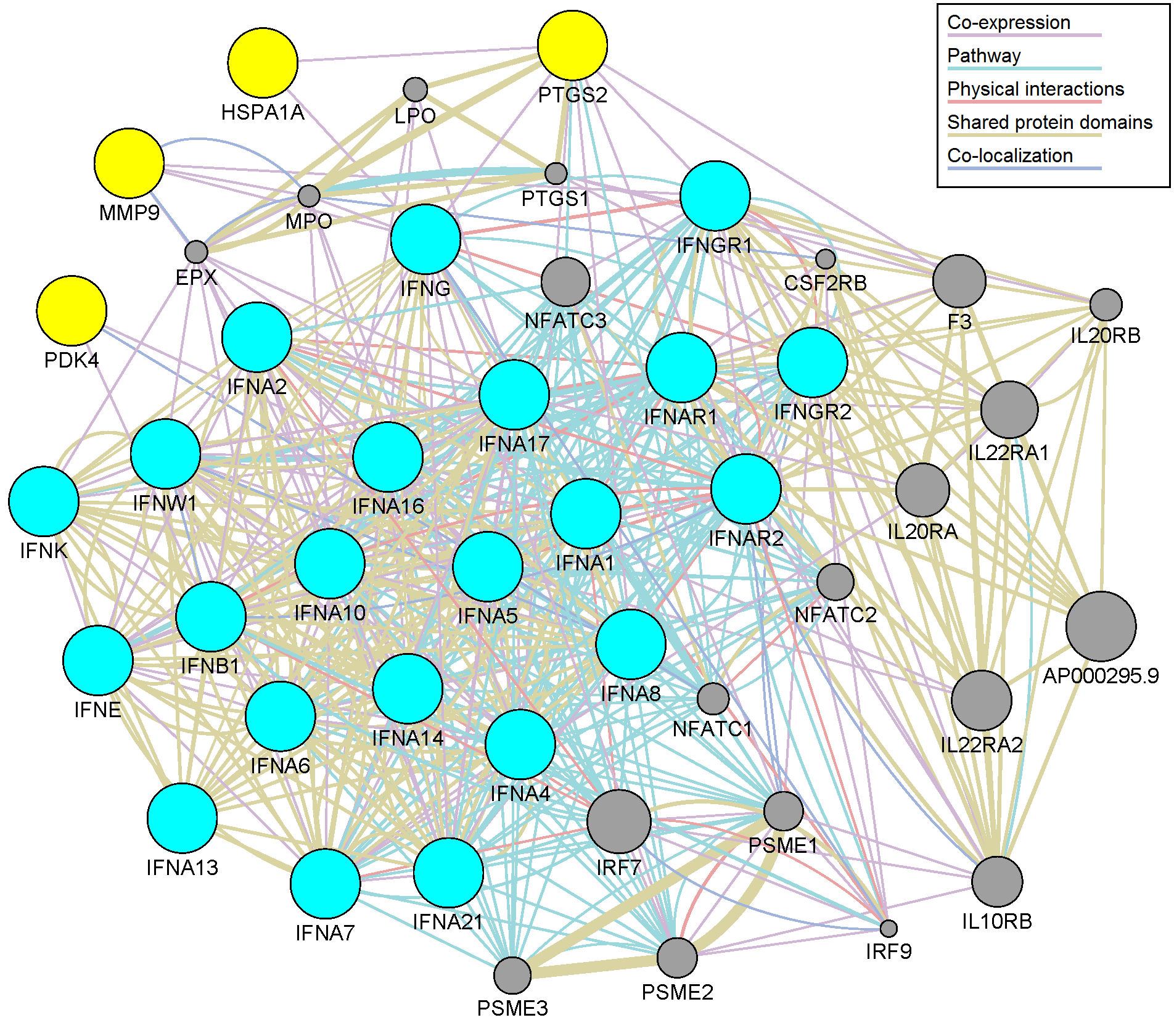


**Supplementary Figure 14.** Connection of interferon signaling genes and anti-stroke target genes in the old group. The cyan cycle represented the interferon signaling genes, and the yellow cycle represented the anti-stroke target genes.


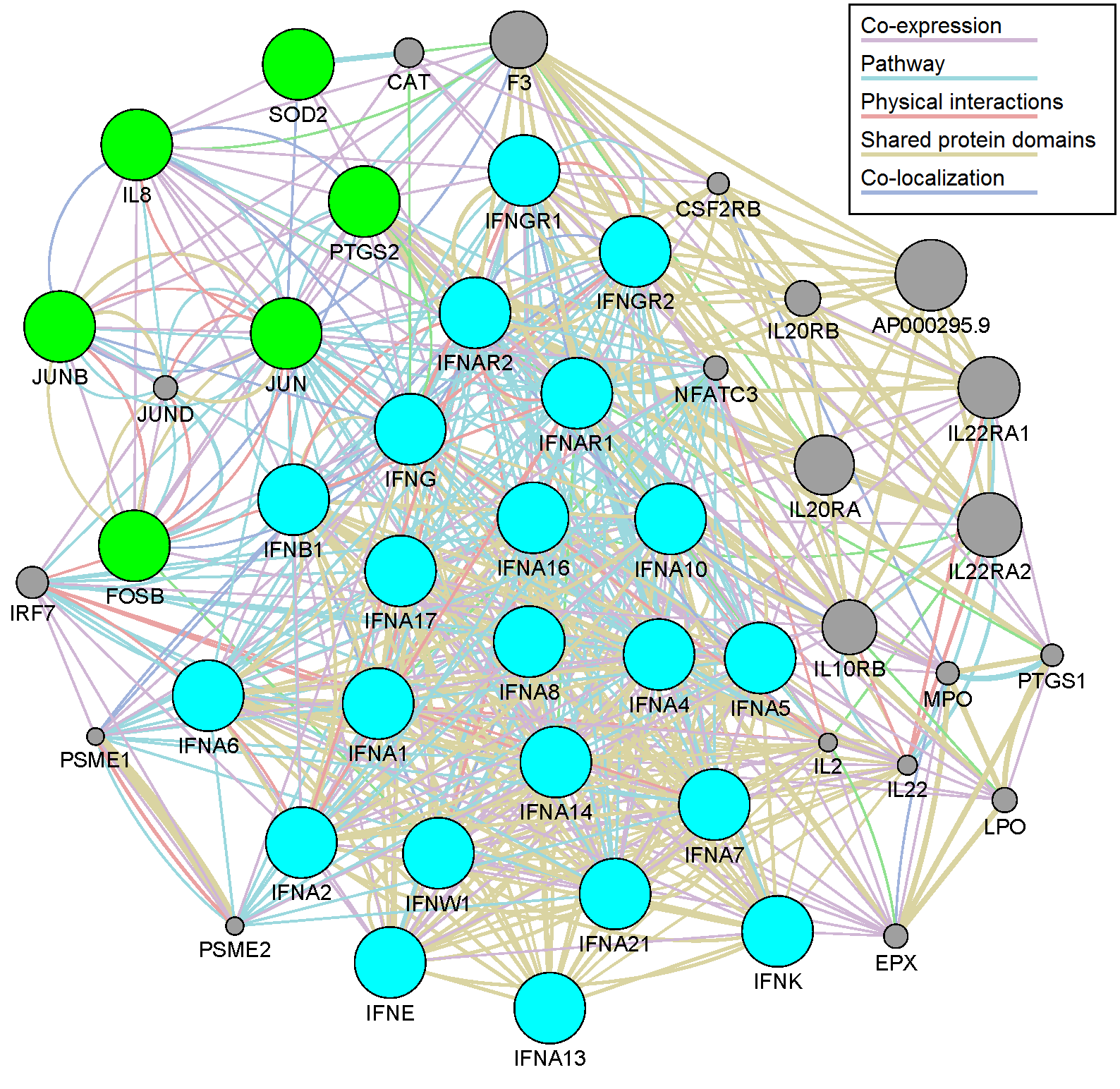


**Supplementary Figure 15.** Connection of interferon signaling genes and anti-stroke target genes in the young group. The cyan cycle represented the interferon signaling genes, and the green cycle represented the anti-stroke target genes.
